# Ultrasonographic assessment of preoperative gastric volume in patients with dyspepsia: a prospective observational study

**DOI:** 10.1186/s12871-021-01559-4

**Published:** 2022-01-12

**Authors:** Yuming Tan, Xianchun Wang, Han Yang, Chuanlong Pan, Nanbo Luo, Junjie Li, Fang Yang, Yanling Bei, Zhen Cahilog, Qian Chen, Zhiheng Liu, Xinping Yang

**Affiliations:** 1 Department of Anaesthesiology, Shenzhen Second People’s Hospital, Shenzhen University First Affiliated Hospital, Health Science Centre, Shenzhen, 518000 China; 2 Department of Ultrasound, Shenzhen Second People’s Hospital, Shenzhen University First Affiliated Hospital, Health Science Centre, Shenzhen, 518000 China; 3 Anaesthetics, Pain Medicine and Intensive Care, Department of Surgery and Cancer, Faculty of Medicine, Imperial College London, Chelsea & Westminster Hospital, London, UK

**Keywords:** Ultrasonography, Gastric antral cross-sectional area, Gastric volume, Dyspepsia, Gastroscopy

## Abstract

**Background:**

Patients undergoing gastroenteroscopy during sedation are prone to aspiration, and most patients with dyspepsia have delayed gastric emptying. This study aimed to investigate the feasibility of measuring the gastric antrum cross-sectional area (CSA) to supply a novel clinical diagnostic reference value in patients with dyspepsia.

**Methods:**

Patients with dyspepsia undergoing elective gastroscopy were included. The Perlas qualitative 0–2 grading scale score was determined before the operation. The anteroposterior diameter (D1) and craniocaudal diameter (D2) between gastric antrum serosal surfaces were measured perpendicular to each other in the supine and right lateral decubitus (RLD) positions. CSA values in the supine position and RLD position were determined. Gastric contents were endoscopically suctioned with the volumes measured and noted as actual gastric volume. Multiple regression analysis was used to fit a mathematical model for estimating the gastric volume. Receiver operating characteristic (ROC) curves were constructed to determine the accuracy of RLD CSA to detect gastric volumes of > 0.8 ml/kg.

**Results:**

A total of 117 patients were enrolled and divided into a functional dyspepsia (FD) group and an organic dyspepsia group according to gastroscopy findings. For a gastric volume of > 0.8 ml/kg, cut-off values for FD and organic dyspepsia were 6.7 cm^2^ and 10.0 cm^2^, respectively. Two new modified mathematical models were derived to predict an estimated gastric volume for FD and organic dyspepsia: volume = 3.93 × RLD CSA - 0.47 × age; and volume = 6.15 × RLD CSA - 0.61 × age.

**Conclusion:**

We used the cut-off value of the antral area for the fast diagnosis of gastric volumes in patients with dyspepsia, which may assist clinicians in identifying patients at risk of aspiration.

**Trial registration:**

www.chictr.org.cn (CHICTR-DDD-17010871); registered 15 March 2017.

## Introduction

Pulmonary aspiration has been extensively recognized since it was reported by Medson [1] in 1946. In the United States, aspiration of gastric contents accounted for 5% of claims in the American Society of Anaesthesiologists (Schaumburg, Illinois) Closed Claims Project database [2]. Although the incidence of pulmonary aspiration is rare, the mortality rate is relatively high once it occurs. In the past, most cases of pulmonary aspiration were deemed to occur during general anaesthesia. Recently, Green [3] et al. presented a valuable comprehensive systematic review of aspiration events during sedation, apparently with a higher risk of gastrointestinal endoscopy reported. The use of sedatives may weaken the protective reflex of the upper respiratory tract. In addition, patients undergoing gastroenteroscopy, most of whom suffer from dyspepsia, may have delayed gastric empties. In patients with functional dyspepsia, delayed gastric emptying, which may be associated with an increased risk for pulmonary aspiration, was found in 20–60% [4].

Previously, there was no well-defined method of identifying patients at risk for aspiration. Pulmonary aspiration was assessed by the anaesthesiologist based on preoperative fasting guidelines recommended by anaesthesia societies. Nevertheless, these fasting guidelines should essentially apply to patients undergoing elective surgery but may not be reliable to patients with delayed gastric emptying [5, 6]. Presently, perioperative ultrasonography of the gastric antrum has proven to be a useful tool for assessing gastric volume. However, previous studies have focused on adults [7], children [8], and pregnant women [9], not people with dyspepsia, who are prone to delayed gastric emptying. The literature has documented that the area of people with dyspepsia fasting gastric antrum is significantly larger than that of healthy adults [10]. Whether earlier models might be equally applied to healthy people and patients with dyspepsia has not yet been determined.

The purpose of this study was to assess the feasibility and predictive value of bedside ultrasound CSA measurements and to supply a novel clinical diagnostic reference value in patients with dyspepsia.

## Materials and methods

### General information

After approval by the Ethics Committee of Shenzhen Second People’s Hospital reviewed and approved this study (No. 20173357201826), we conducted a prospective observational study registered at the Chinese Clinical Trial Registry (CHICTR-DDD-17010871). All methods were carried out in accordance with relevant guidelines and regulations. The reporting of this study conformed to the STROBE statement [11]. For this study, 120 patients underwent elective gastroscopy in the hospital from June 2018 to June 2019. Their age was ≥18 years, American Society of Anaesthesiologists physical status I to II, and body mass index (BMI) B < 35 kg/m^2^. They complained of upper abdominal discomfort or pain, full stomach after meals, or symptoms of bloating or early satiety, accompanied by inappetence, belching, nausea, or vomiting [12]. Patients with clinical manifestations of dyspepsia were diagnosed by an attending physician who had more than 10 years of clinical practice. All patients were classified by their physicians as having FD or organic dyspepsia. Organic dyspepsia was defined as oesophagitis, peptic ulcer disease, erosive gastritis, benign oesophageal stricture, Barrett oesophagus, or upper gastrointestinal malignancy [13]. All other findings at endoscopy were classified as functional dyspepsia. A diagnosis of FD was made based on a minimum history of 3 months of nausea, abdominal pain, and/or discomfort referred to the upper abdomen after exclusion of organic causes [14]. They voluntarily asked for a painless or normal gastroscopy to be performed after an 8-h fasting and a 2-h of no drinking before surgery to prevent gastroscopy complications. The following exclusion criteria were adopted for patients: a previous history of upper gastrointestinal surgery; upper gastrointestinal anatomical abnormalities (oesophageal hiatal hernia); upper gastrointestinal bleeding; liver disease; gallbladder disease; patients who did not undergo gastric ultrasound scans or gastroscopy; and pregnant women.

### Study method

**Fig. 1:**
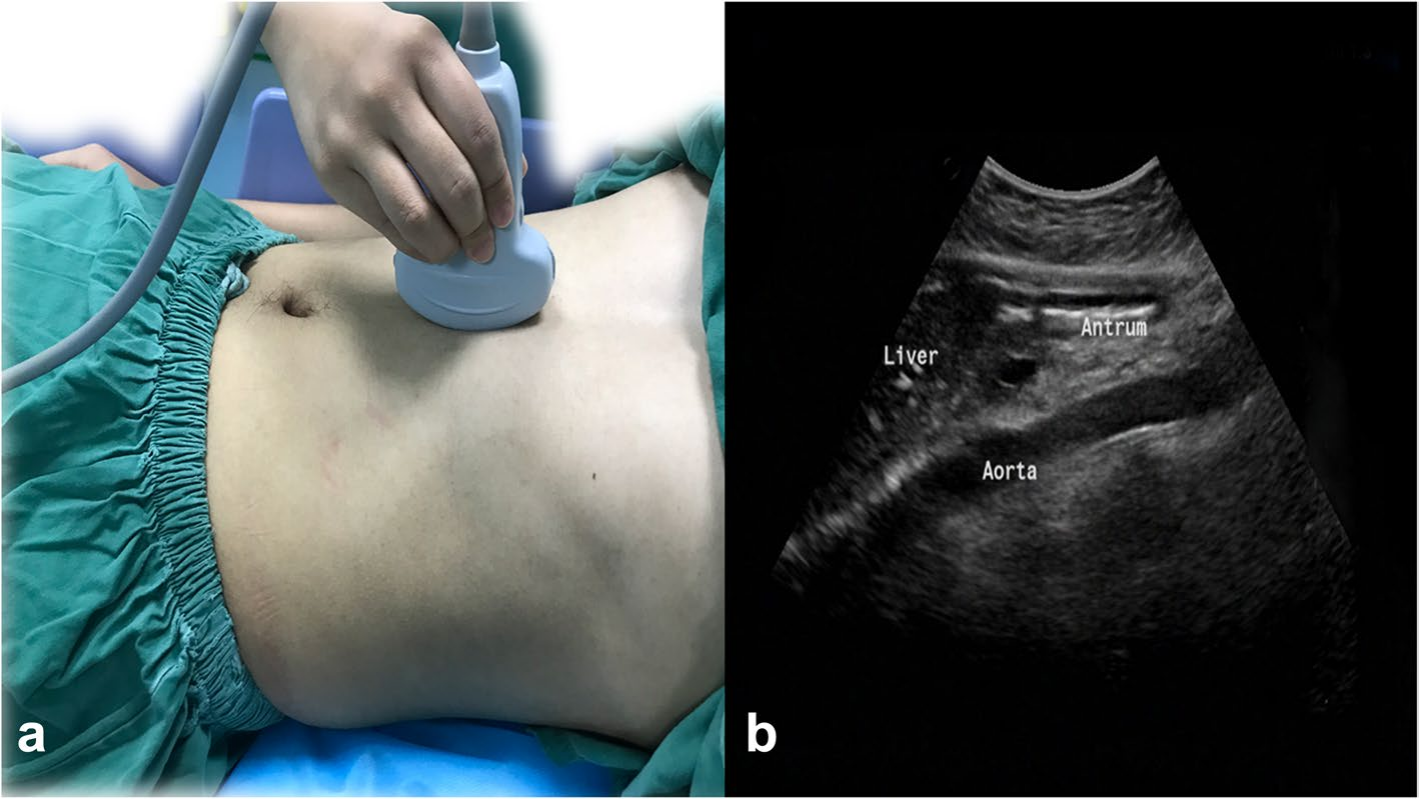
Transducer position to scan the gastric antrum (**a**). Antral ultrasound image (**b**)

After admission to the operating room, ultrasonography was performed by trained personnel using a colour Doppler ultrasound system (Model M-Turbo, probe frequency 2–5 MHz, transducer C60x, Sonosite, abdominal imaging mode, United States). Each ultrasound examination was performed with the first patients’ heads raised at 45° in the supine position and then in the right lateral decubitus (RLD) position. The sagittal or parasagittal plane of the upper abdomen was scanned using the left hepatic lobe, abdominal aorta, inferior vena cava, or superior mesenteric veins/arteries as anatomical landmarks (Fig. 1) [15]. The gastric antrum was located to determine the nature of the gastric contents to record them as air and/or liquid and/or solid and to grade the patients according to Perlas [7] three-point qualitative grading system: grade 0, no stomach contents visible in the supine and RLD positions; grade 1, no stomach contents visible in the supine position but becoming visible as liquids in the RLD position; and grade 2, gastric fluid visible in both supine and RLD positions. In our study, solid food detected in any position was rated as grade 2; hence, gastric volume estimation was not performed. The maximum anteroposterior diameter (recorded as D1) and maximum craniocaudal diameter (recorded as D2) between the gastric antrum serosal surfaces were measured perpendicularly to each other. Three measurements were taken, and the average values of D1 and D2 were used to calculate the cross-sectional area (CSA) of the patient’s gastric antrum in the supine and RLD positions using the formula described by Bolondi [16]: CSA(cm2) = (π × D1 × D2) / 4.

The formula of Perlas et al. [17], which was developed in adults to predict gastric volumes based on the cross-sectional area, was applied as follows: gastric volume (Vol, in ml) = 27.0 + 14.6× RLD CSA (cm^2^)-1.28 × age (years).

Thereafter, the patients underwent gastroscopy in the left lateral decubitus position. Gastroenterologists were blind to the results of the preceding ultrasound scans. All the stomach contents were aspirated, and the volume was measured and used as the actual gastric volume.

The patients’ age, sex, weight, height, BMI, CSA in two positions (supine position and RLD position), actual stomach volume, and diagnosis of subsequent gastroscopy were also recorded.

After the above investigations, the patients were divided into two subgroups: organic dyspepsia and FD. Patients were included in the organic group if they were diagnosed with organic dyspepsia and in the FD group if they were diagnosed with functional dyspepsia (FD) by their assessing gastroenterologist. Six-three patients were in the group with organic dyspepsia, 54 patients were in the FD group.

### Statistical methods

SPSS 24.0 was used for statistical analyses and to create receiver operating characteristic (ROC) curves of ultrasonographic diagnosis of a gastric volume > 0.8 ml/kg. This study referred to a method adopted in a previous study [18] with a sensitivity of 0.91, accuracy of 0.1, and predictive value of 0.63. The diagnostic test sample included 51 people, and based on the sample shedding rate, *N* = 120 people were included.

Normally distributed measurement (parametric) data are expressed as the mean ± standard deviation (± SD), and independent group t-test and analysis of variance (ANOVA) analyses were used for group comparisons. Nonnormally distributed measurement (nonparametric) data were expressed as median and interquartile ranges (Q25, Q75), and the Kruskal–Wallis H test was used for group comparisons. Classification data (sex, reflux oesophagitis, gastric mucosal erosion, gastric ulcer) are expressed as incidence or % ratios, and chi-square analysis and Fisher’s exact test were used for group comparisons. Spearman’s correlation analysis was employed to analyse the relationship between the CSA of the gastric antrum and the actual gastric volume in the supine and RLD positions for the organic group and FD group, respectively. The intraclass correlation coefficient (ICC) was employed to test the consistency of the predictive ability of the Perlas model and our present model for the organic group and FD group, respectively. Multiple linear regression analysis served to establish our new model for the organic group and FD group. The ROC curve was plotted to evaluate the diagnostic power of the gastric antrum areas in the RLD position in predicting gastric volume > 0.8 ml/kg for the organic group and FD group. The area under the curve (AUC), sensitivity, specificity, and Youden index were calculated at the cut-off value of the ultrasound measurements of the gastric antrum areas in the RLD position. The grey zone was constructed to eliminate uncertainty about the range of values that predict gastric volume > 0.8 ml/kg. The grey zone was defined as an area between the cut-off values, with the minima in which the sensitivity or specificity was below 90%. A value of *P* < 0.05 was considered statistically significant.

## Results

### Patient characteristics

In this study, 120 patients with dyspepsia were recruited. Three patients were excluded due to excessive gas in the stomach and inability to display a complete antrum section under ultrasound. Thus, a total of 117 subjects (42 males and 75 females) were enrolled and analysed. Table 1 sums up the patients demographic. Forty patients had Perlas grade 0, sixty-eight patients had Perlas grade 1, and nine patients had Perlas grade 2. Of the grade 2 patients, 8 patients had solid contents in the antrum. Among the gastroscopy findings, reflux oesophagitis accounted for 7.7% of the diagnoses, gastric mucosal erosion accounted for 30.8%, and gastric ulcer accounted for 15.4%. There were statistically significant (*P* < 0.05) differences in gender, incidence of gastric mucosal erosion, and incidence of gastric ulcer between groups, but none for age, weight, BMI, incidence of gastroesophageal reflux, or incidence of FD. No episodes of aspiration were recorded.

**Table 1:** Patient characteristics by gastric antrum grade

	Perlas grading			Study object (N total = 117)	*p*
	Grade 0 (*n* = 40)	Grade 1 (*n* = 68)	Grade 2 (*n* = 9)		
Male:female	8/32	28/40	6/3	42/75	0.01
Age	43.2 (31.3–55.0)	40.9 (28.0–50.0)	40.2 (25.5–56.0)	41.6 (29.5–54.0)	0.65
BMI (kg/m)	22.3 (20.0–24.1)	22.3 (20.1–24.2)	22.8 (19.7–26.5)	22.3 (20.1–24.2)	0.81
Weight (kg)	58.4 (50.5–64.5)	61.3 (53.0–67.8)	64.4 (54.0–71.5)	60.6 (52.5–66.0)	0.23
Reflux Oesophagitis	5 (12.5%)	4 (5.9%)	0 (0.0%)	9 (7.7%)	0.11
Gastric ulcer	2 (5.0%)	12 (17.6%)	4 (44.4%)	18 (15.4%)	< 0.01
Gastric mucosal erosion	18 (45.0%)	18 (26.5%)	0 (0.0%)	36 (30.8%)	0.03
FD	15(37.5%)	34(50.0%)	5(55.6%)	54(46.2%)	0.17

Data represent n (%), ratio, or median and interquartile ranges (IQR 25–75%), *BMI* body mass index, *FD* functional dyspepsia

### Comparison of the gastric antrum cross-sectional area, predicted gastric volume, and actual gastric volume in various decubitus positions

According to the aetiology and gastroscopy findings, dyspepsia was divided into 2 groups: organic dyspepsia and FD. The presence of solid gastric contents in 8 subjects was excluded from the gastric fluid-volume calculation (FD: *n* = 5; organic dyspepsia: *n* = 3). The gastric antrum CSA in the RLD position, the predicted gastric volume, the actual measured gastric volume, and the measured gastric volume per unit body weight of the patients in different Perlas grades were significantly different (*p* < 0.05), with a positive correlation noted in both groups. As shown in Tables 2 and 3, the CSA of the gastric antrum in the supine position did not have a statistically significant difference among Perlas grades in either subgroup. Furthermore, the area of the gastric antrum in the RLD position was moderately correlated with the measured gastric volume in patients with FD (Spearman rank correlatio*n* = 0.47, *p* < 0.001), but a significant correlation was found in the patients with organic dyspepsia (Spearman rank correlation = 0.61; *p* < 0.0001).

**Table 2:** Ultrasound-measured antral area and gastric volume by the gastric antral grades of FD

	Grade 0 (*n* = 15)	Grade 1 (*n* = 34)	Grade 2 (*n* = 0)	*p*
Supine position CSA (cm^2^)	3.9(3.0, 4.5)	4.2(3.2, 4.7)	0	0.333
RLD CSA (cm^2^)	6.1(4.5, 7.7)	8.9(6.7, 10.7)	0	< 0.0001
Actually measured volume (ml)	15(8.0, 18.0)	45(29.0, 52.5)	0	< 0.0001
Predicted gastric volume (ml)	57.9(47.8, 118.1)	109.1(75.0, 139.0)	0	< 0.0001
Gastric volume/unit body weight (ml/kg)	0.2(0.1, 0.4)	0.7(0.5, 0.9)	0	< 0.0001

Volumes and antral cross-sectional areas (CSAs) are presented as median and interquartile ranges (IQR 25–75%), *RLD* right lateral decubitus, *CSA* cross-sectional area

**Table 3:** Ultrasound-measured antral area and gastric volume by the gastric antral grades of organic dyspepsia

	Grade 0 (*n* = 25)	Grade 1 (*n* = 34)	Grade 2 (*n* = 1)	*p*
Supine position CSA (cm^2^)	4.9(3.7, 5.7)	4.6(3.7, 5.3)	4.7	0.825
RLD CSA (cm^2^)	6.9(5.7, 8.2)	10.6(7.9, 12.1)	12.0	< 0.0001
Actually measured volume (ml)	12.0(6.0, 15.5)	33.5(29.5, 61.0)	150.0	< 0.0001
Predicted gastric volume (ml)	70.0(53.7, 87.6)	115.9(90.2, 136.8)	177.4	< 0.0001
Gastric volume/unit body weight (ml/kg)	0.2(0.1, 0.3)	0.6(0.5, 1.0)	2.8	< 0.0001

Volumes and antral cross-sectional areas (CSAs) are presented as median and interquartile ranges (IQR 25–75%), *RLD* right lateral decubitus, *CSA* cross-sectional area

### Receiver operating characteristic (ROC) curve

**Fig. 2:**
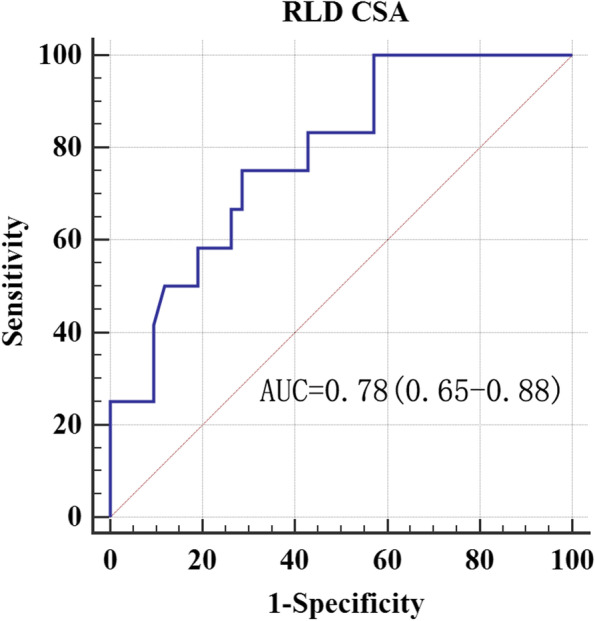
ROC curve of gastric antrum CSA in patients with FD. Receiver operating characteristic curves for the detection of a gastric volume greater than 0.8 ml/kg by the measurement of antral area in the RLD positions. AUC = area under the receiver operating characteristic curve with 95% confidence interval. *P* = 0.0001

**Fig. 3:**
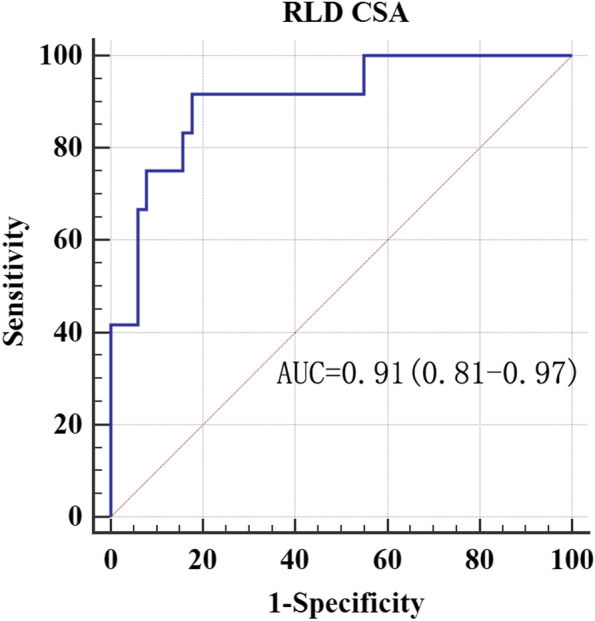
ROC curve of gastric antrum CSA in patients with organic dyspepsia. Receiver operating characteristic curves for the detection of a gastric volume greater than 0.8 ml/kg by the measurement of antral area in the RLD positions. AUC = area under the receiver operating characteristic curve with 95% confidence interval. *P* = 0.0001

The ROC curves obtained for the ultrasonographic diagnosis of a gastric volume of 0.8 ml/kg are shown in Figs. 2 and 3. At the optimal cut-off value of the RLD CSA for each ROC curve, the ultrasonographic measurements of the RLD CSA are summarized in Table 4. The cut-off value for patients with FD to predict gastric volume of > 0.8 ml/kg was 6.7 cm^2^, with a sensitivity of 100% and a specificity of 48.7%. The cut-off value for patients with organic dyspepsia to predict gastric volume > 0.8 ml/kg was 10.0 cm^2^, with a sensitivity of 92. 7% and a specificity of 82.4%.

**Table 4:** Ultrasonographic measurement of antral CSA for preoperative diagnosis of gastric volume > 0.8 ml/kg

	AUC	Threshold value (cm^2^)	Sensitivity	Specificity	Youden index
FD	0.78	6.7	100	48.7	0.5
Organic dyspepsia	0.91	10.0	91.7	82.4	0.7

*FD* functional dyspepsia

**Fig. 4:**
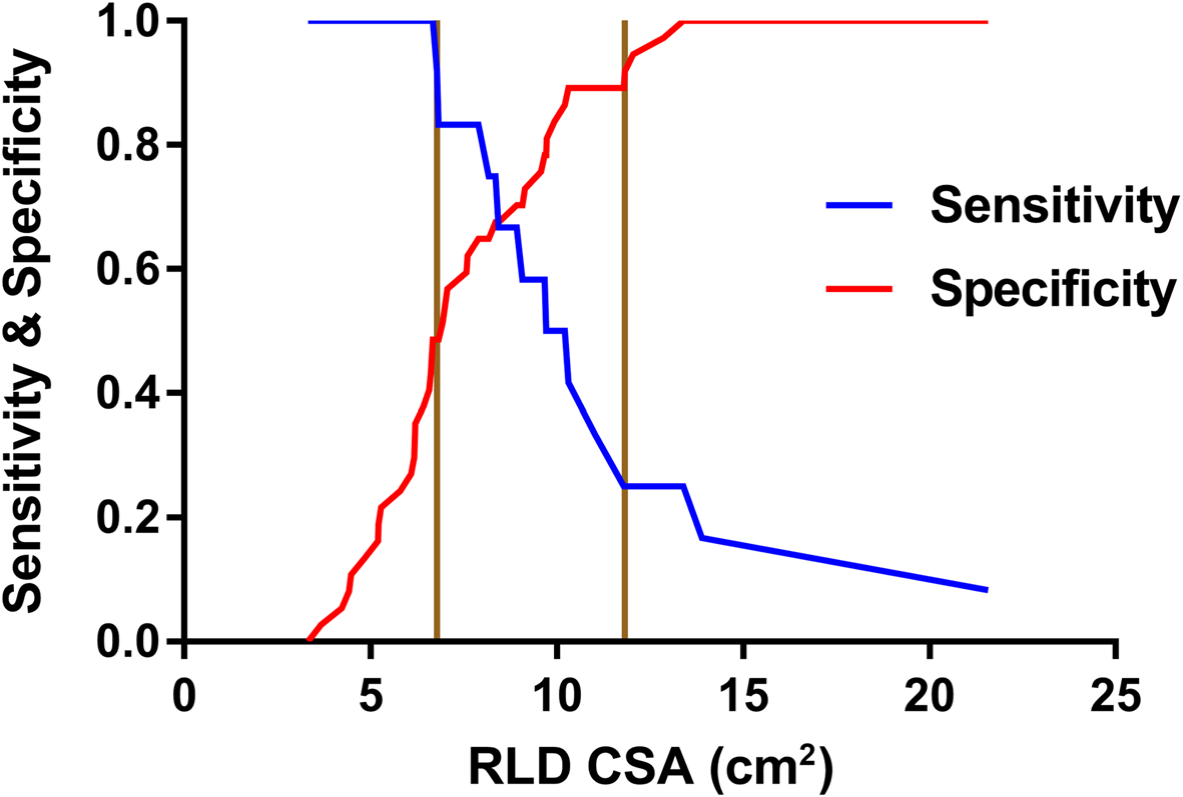
Grey zone approach indicates two cut-offs, between which the diagnosis of a gastric volume greater than 0.8 ml/kg by the measurement of antral area in the RLD positions in patients with FD remains uncertain

**Fig. 5:**
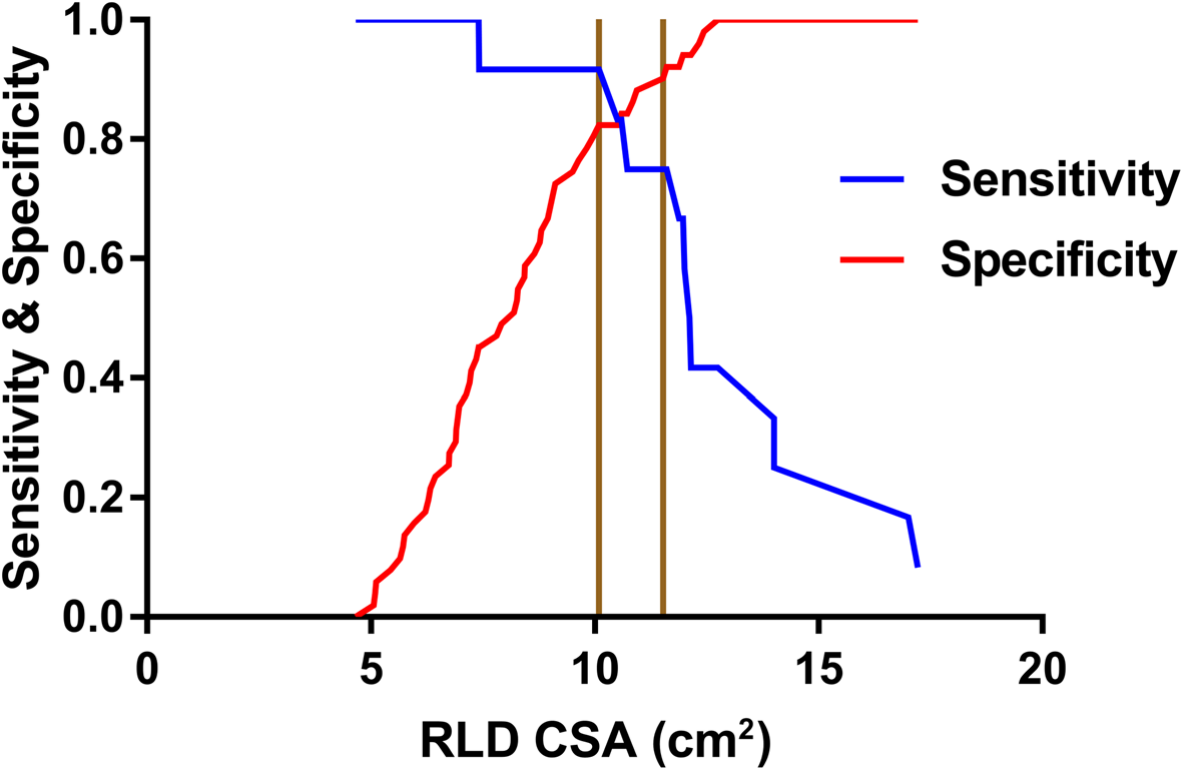
Grey zone approach indicates two cut-offs, between which the diagnosis of a gastric volume greater than 0.8 ml/kg by the measurement of antral area in the RLD positions in patients with organic dyspepsia remains uncertain

The grey zone approach identified score ranges and numbers of patients in the grey zone for diagnosis with indeterminate risk as follows: 6. 8 to 11.8 and 23 (47%) patients in the FD group (Fig. 4); 10.1 to 11.5and 5 (8%) patients in the organic dyspepsia group (Fig. 5).

### Reconstructed model of predicted gastric volume after correction

We used the mathematical model of the relationship between gastric antrum CSA and the actual gastric volume as established according to Perlas et al. in a study of adults to predict the actual gastric volume of patients with dyspepsia. The results showed that the consistency between the two groups of subjects was poor (ICC < 0.4). Based on the original mathematical model, the linear relationship was measured for each group between the independent variable and the dependent variable. Seven independent variables, including age, sex, height, weight, BMI, supine CSA, and RLD CSA, were rescreened using forward regression and obtained the corrected mathematical model (Table 5). Next, the consistency between the predicted gastric volume and the actual gastric volume was analysed. The results showed that the model consistency after correction was improved (ICC of patients with FD = 0.63 and ICC of patients with organic dyspepsia = 0.67).

**Table 5:** Multiple linear regression model for the prediction of gastric volume

	Mathematical model	Adjust R^2^
FD	Volume ml = 3.93 × RLD CSA (cm^2^) -0.47 × age	0.40
Organic dyspepsia	Volume ml = 6.15 × RLD CSA (cm^2^) -0.61 × age	0.49

*FD* functional dyspepsia

## Discussion

The main finding of our study was the calculation of gastric volume in patients with dyspepsia and setting of a gastric antral threshold to rapidly assess the risk of pulmonary aspiration by ultrasound. Through our study, we note that although fasting guidelines had been observed, the incidence of a full stomach in patients with dyspepsia was 7.7% (9/117). In patients found to have a full stomach, anaesthetic management was changed to prevent aspiration. It is of great clinical importance to be able to quickly and accurately assess the risk of pulmonary aspiration during elective gastroscopy in patients with dyspepsia. As expected, higher antral grades were associated with larger antral CSA and greater gastric volume, similar to previous adult studies [7]. Interestingly, we found that the incidence of gastric ulceration also increased with antral grade.

Our data supported that the median gastric antrum CSA in the RLD position for grade 0 to 1 was greater than that previously reported in adults [7] and pregnant patients [9] (FD: 6.1 cm^2^, organic dyspepsia: 6.9 cm^2^, adult: 3.6 ± 1 cm^2^, pregnant women: 4.2 ± 1.6 cm^2^ for grade 0; FD: 8.9 cm^2^, dyspepsia: 10. 6 cm^2^, adult: 5.6 ± 1.4 cm2, pregnant women: 5.7 ± 2.1 cm^2^ for grade 1). These results were consistent with previously reached conclusions in patients with non-ulcer dyspepsia [10, 19]. This increase in the gastric antrum area may be due to altered interdigestive gastroduodenal motility characterized by the absence of gastric activity fronts (phase III) [20]. The lack of phase III of the migrating motor complex (MMC) impairs the stomach’s ability to evacuate its content, thus causing a significantly decreased gastric antrum frequency and amplitude of contractions in patients with dyspepsia. This suggested that such patients have gastrointestinal motility impairment during digestion, which is therefore likely to be the cause of the larger gastric antrum area during fasting. By combining these factors, we concluded that the mathematical model set up in adults could not precisely predict the gastric volume of patients with dyspepsia. Therefore, we presented the first mathematical model to predict gastric volumes for patients with dyspepsia.

In the current study, the CSA in the RLD position moderately correlated with the volume, while the CSA of the gastric antrum in the supine position poorly correlated with the volume. Perlas et al. [21] demonstrated that the linear relationship between the gastric volume and the RLD position is superior to the supine position, which may be explained by the fact that gravity causes gastric fluid to fill the gastric antrum as the subject moves to the RLD position. In addition, there was a poor significant correlation between CSA in the supine position and gastric volume, which may be related to the larger area of gastric antrum in patients with fasting. As previously mentioned, the gastric wall of the subjects in our cohort did not contract to the corresponding gastric antrum area when the gastric volume was low, which may more strikingly impact the correlation between CSA and gastric volume in the supine position than in the RLD position.

We derived two novel linear regression formulas to estimate the predicted gastric volume according to the CSA in the RLD position and the subjects’ age. Published literature [18, 21] reveals that many demographic and anthropometric factors displayed strikingly correlated with gastric volume. Nonetheless, demographic variables (sex, height, weight, and BMI) and CSA in the supine position were not found to be independent predictors of gastric volume in our study. ICC was performed to verify the accuracy of the formula showing improved consistency. Of note, an ICC coefficient value of ≥0.7 indicates good consistency [22], while a value range from 0.55–0.69 was usually considered moderate agreement, revealing relevant imprecision. Consequently, this might lead to questions on the applicability of such models in clinical practice.

Previous literature has documented that various mathematical models for predicting gastric volumes have a high correlation coefficient, with adjusted regression R2 values ranging from 0.6–0.73 [17, 18]. However, the value of the current study, i.e., adjusted R^2^ = 0.40 in FD and adjusted R^2^ = 0.49 in organic dyspepsia, was lower than that observed by Perlas and Bouvet et al. Although our cohort population has a high incidence of delayed gastric emptying, which means that the distribution of the number of gastric antrums in grade 2 was high, the actual number of patients with delayed gastric emptying was significantly less than that of the previous literature reporting a prevalence ranging between 20 and 50%. In addition, when the full stomach contained solid food, the success rates of imaging a complete cross-section were low by Sonography [21]. The solid/air mixture presented as mixed echogenicity by ultrasound forms an artefact obscuring the posterior wall of the antrum. Further, patients with grade 2 antrums mostly contained solid food, and their actual gastric volume could not be aspirated due to larger solid particles blocking the gastroscope tube, which introduced bias or errors into the actual gastric volume collection. Therefore, the low adjusted regression R2 can be ascribed to insufficient sample size and invalid data of patients with grade 2 antrum. Our prediction model needs to be modified by incorporating a larger sample size.

Considering the 91.7% sensitivity and 82.4% specificity of 10.0 cm2 as the cut-off value of antral RLD CSA, we recommend using this value to diagnose a gastric fluid volume > 0.8 ml/kg in patients with organic dyspepsia. Nevertheless, the cut-off value of the patients with FD did not have excellent specificity as patients with organic dyspepsia. Therefore, this result may not be suitable for clinical practice. Our results showed a higher AUC in patients with organic dyspepsia than in patients with FD. Whether insufficient computable data in patients with grade 2 or pathological features in patients with functional dyspepsia causing this result is unidentified temporarily. For Perlas grade 2, the FD subgroup had invalid data, whereas organ dyspepsia has only one patient computable data. The gastric antrum expands as the gastric volume increases within a specific limit [21]. Patients with dyspepsia have contractibility of antrum dysfunction at low gastric volume. Still, its dilatation function remained regular, meaning that accuracy in the ability of gastric antrum diagnosis increased with the number of patients with grade 2 gastric antrum. However, only one calculation data cannot explain the higher AUC in the case of organic dyspepsia. We know that this result is in accordance with the finding of the Spearman rank correlation.

The grey zone is defined as a quantitative test, and the biological indicators falling into this area can neither diagnose nor exclude the disease. A test result falling in the grey zone is not uninformative, as it could overcome the shortcoming of the ROC curve of using a single cut-off value and remind clinicians to use additional tools to diagnose disease. A previous study revealed that the data-driven choice of cut-off tends to exaggerate the diagnostic performance of the biomarker [23]. Identifying the proportion of results that will fall within the grey zone will also help assess the usefulness of a test in practice [24]. In our study, a wide range of grey zone values and nearly half the patients with FD were in the grey zone, showing the poor discrimination ability of the diagnosis of a gastric volume greater than 0.8 ml/kg by the measurement of antral area in the RLD positions. Conversely, a narrow range of grey zone values and a lower proportion of patients with organic dyspepsia were in the grey zone, denoting the good discrimination ability of the test.

The threshold values of aspiration risk have been constantly controversial. An animal study demonstrated that an acidic gastric volume > 0.4 ml/kg produced pulmonary aspiration using direct instillation of the monkey’s lungs [25]. If these results were to be extrapolated to humans, the critical volume for severe aspiration could be increased from 25 ml to 50 ml (0.8 ml/kg) [26]. Previous studies have shown that the residual volume in healthy volunteers can range from 0 to 115 ml with a mean range of 25–27 ml after an overnight fast [27, 28]. Perlas suggested that a critical gastric fluid volume > 1.5 ml/kg in healthy people might increase aspiration risk [7]. However, neither animal nor human studies identified the validity of extrapolating from one volume to the other, which needs to be clarified in further studies. In our study, we chose a strict threshold of gastric volume > 0.8 ml/kg for determining the risk of aspiration due to a combination of all risk factors for aspiration, including gastrointestinal problems, airway problems, inadequate depth of anaesthesia, and depressed consciousness. According to the data [29] of the gastrointestinal endoscopy survey conducted in China, the highest incidence of related complications was aspiration. Nevertheless, deep intravenous sedation with propofol and fentanyl without tracheal intubation was performed routinely. Based on all these findings, we cautiously used strict thresholds.

We have to acknowledge some limitations of our study. The fasting duration was not recorded in our cohort. Some patients have exceeded the 8-h fasting time due to gastrointestinal symptoms, and the stomach volume might change significantly. As the cohort of patients in our study required fasting, the number of patients with grade 2 included in both functional and organic dyspepsia subgroup was insufficient. Therefore, our presently proposed mathematical model had a bias. This is a descriptive observational single-cohort study lacking a control group, and the results must be evaluated in the context of the study.

In conclusion, we propose that for patients with organic dyspepsia, cut-off values of 10.0 cm2 should be measured antral cross-sectional area in right lateral decubitus by ultrasound to detect gastric volumes> 0.8 ml/kg. Further research is warranted to fully determine the threshold of the antral cross-sectional area for patients with functional dyspepsia. Our presented mathematical model is not enough to accurately predict gastric volume for clinical practice. However, ultrasonography continues to play an important role in guiding management, particularly when it is difficult to determine the risk of aspiration in clinical practice.

## Data Availability

All data associated with this study are present in the paper or the supplementary materials and are available upon request from the corresponding authors.

## Abbreviations

CSA: Cross-sectional area
RLD: Right lateral decubitus
FD: Functional dyspepsia
ROC: Receiver operating characteristic
AUC: Area under the curve
ASA: American Society of Anaesthesiologists
BMI: Body mass index
ANOVA: Analysis of variance
ICC: Intraclass correlation coefficient
IQR: Interquartile range
MMC: Migrating motor complex
